# Effects of Potassium Fulvic Acid on Soil Physical and Chemical Properties and Soil Microenvironment of Blueberry (*Vaccinium corymbosum* L.) Under Salt Stress

**DOI:** 10.3390/plants14111654

**Published:** 2025-05-29

**Authors:** Xuanrong Wu, Dekang Hou, Jing Ma, Yanan Li, Lin Wu, Haiguang Liu, Yi Zuo, Xinxin Guo, Jinying Li, Ying Wang

**Affiliations:** College of Horticulture, Jilin Agricultural University, Changchun 130118, China; wxr17780829960@163.com (X.W.); hdk131476431192022@163.com (D.H.); 13674498905@163.com (J.M.); liyanan@jlau.edu.cn (Y.L.); linw@jlau.edu.cn (L.W.); haiguangl@jlau.edu.cn (H.L.); m15087108512@163.com (Y.Z.); gxx101315@outlook.com (X.G.)

**Keywords:** blueberry, potassium fulvic acids, salt stress, soil physicochemical properties, soil enzyme activity

## Abstract

These days, one of the main issues preventing agricultural development is salinized soils. Potassium fulvic acid (PFA) not only regulates plant growth, but also improves the soil nutrient content and physical structure, which makes it a soil conditioner worth promoting. Nevertheless, the research conducted thus far on the subject of PFA with regard to plant growth and inter-root microbial communities remains somewhat limited in scope. In this study, a pot experiment was conducted to simulate both the normal environment and salt stress environment. The objective of this experiment was to verify the effect of PFA on the growth of blueberry (*Vaccinium corymbosum* L.) as well as its effect on the soil physical and chemical indices and the soil microbial community structure. The findings demonstrated that the implementation of potassium fulvic acids exhibited a minimal impact on the growth of blueberry plants under standard environmental conditions. However, it was observed to exert a substantial effect on enhancing various physiological parameters, including plant height, root activity, and chlorophyll synthesis, particularly in response to salt stress. PFA led to a substantial augmentation in the soil organic matter content, alongside a notable rise in the alkali-hydrolyzable nitrogen (AN) and available potassium (AK) content. Concurrently, PFA caused a notable escalation in the activities of soil urease, sucrase, acid phosphatase, and catalase (*p* < 0.05) in the salt-stressed environment. PFA increased the abundance of Acidobacteria, Myxococcota, Ascomycota, and Fungi_phy_Incertae_sedis under salt stress, which was mainly related to the decrease in electrical conductivity (EC) values and increase in soil acid phosphatase (S-ACP) activity. It is evident that the implementation of PFA is advantageous in enhancing the saline environment, mitigating the impact of salt damage on blueberries and establishing a foundation for the expansion of cultivated areas and the sustainable cultivation of blueberries.

## 1. Introduction

When soluble salts amass persistently in soil, the process is termed soil salinization and includes primary salinization (natural process) and secondary salinization (artificial induction) [1,2]. Among them, secondary salinization mainly includes agricultural farming [3], road salting [4], and oil exploitation [5]. Human activities can elevate the background soil salinity to phytotoxic levels, exacerbating global soil salinization. Such environmental degradation severely constrains agricultural productivity [6,7,8]. It is estimated that the global area of saline land will exceed 1.381 × 10^9^ hectares (hm^2^) by 2024, with an annual increase of 1.0 × 10^6^–1.5 × 10^6^ hectares (hm^2^) per year [9,10,11]. The increasing area of salinized soil has gradually threatened the yield and quality of food and fruits worldwide, and the improvement in salinized soil has become an urgent problem. The area of saline soil in China has been estimated to be approximately 3.69 × 10^7^ hm^2^, which constitutes approximately 5.01% of the total available land area in the country. It is one of the most widely distributed countries in the world, mainly distributed in northeast, northwest, and coastal areas of China [12,13]. In recent years, the control measures for salinized soil have mainly included physical, chemical, and bioremediation methods. A series of improvements and treatments have been carried out on existing salinized soil, and some achievements have been made in improving the environment of salinized soil [14]. Blueberry(*Vaccinium corymbosum* L.) belongs to Ericaceae and Vaccinium, which is an emerging world small berry fruit tree [15,16]. In 1908, the United States Department of Agriculture took the lead in the artificial domestication and cultivation of blueberries. To date, there are more than 30 main varieties of blueberries in six major producing areas in the world [17]. Blueberry was introduced into China in the 1980s. By 2024, the area planted with blueberries in China will reach 9588 hm^2^, and total production will reach 780,000 tons [18]. In recent years, the cultivation of blueberry has achieved considerable economic benefits and played an important role in rural revitalization and rural economic development. However, there are a large number of salinized soils in the coastal and northwestern regions of China, which seriously limit the growth of blueberries and the expansion of cultivation areas. 

Fulvic acid (FA) is a small molecule organic compound in humic acid (HA). Potassium fulvic acid (PFA) is an organic compound that functions as a potassium fertilizer. The production of this compound entails the introduction of potassium ions into the acidic functional group of fulvic acid. It is readily soluble in acidic environments and is efficiently absorbed by plants. It is very suitable for plants growing in acidic environments such as blueberry [19,20,21]. Potassium fulvic acid has been demonstrated to enhance soil porosity, facilitate the adsorption and stabilization of metal cations through the chelation and binding of active groups, reduce the soluble salt concentration of soil, and mitigate the degree of soil salinization [22,23,24]. PFA can absorb and store K^+^, reduce the amount of K lost due to water, avoid the fixation of K by clay soil, and increase the amount of exchangeable K [25]. In addition, PFA can also promote improvements in the soil micro-ecological environment and improve plant resistance to abiotic stress, thus promoting plant growth and development [23,26,27].

The rhizosphere is a narrow soil area that surrounds the plant roots. Various microorganisms, such as bacteria, fungi, archaea, nematodes and algae, are found in the rhizosphere. These rhizosphere soil microorganisms have direct or indirect effects on plant growth and nutrient absorption, and also have the effect of regulating soil nutrient structure and improving soil enzyme activity [27,28,29]. Soil microorganisms can improve the fertility of saline-alkali land through nitrogen fixation and the transport of key nutrients to crop plants, thereby improving the absorption and transformation of nutrients by plants and also regulating the soil structure [30]. For example, Wang et al. [31] reported that irrigating plant growth-promoting rhizosphere (PGPR) strains in the rhizosphere of tea plants significantly increased the content of organic carbon, nitrogen, phosphorus, and potassium in soil, thereby promoting further growth in tea plants. In addition, Li et al. [32] found that the uptake and utilization of soil mineral elements in blueberry cultivation were closely related to the structure of the soil bacterial microbial community and that this relationship promoted the formation of mycorrhizal roots with blueberry roots, thereby facilitating the uptake of soil mineral elements and nutrients. Studies have shown that ericoid mycorrhizal (ERM) bacteria isolated from wild blueberry roots can promote the absorption of nitrogen and phosphorus in the environment [33]. Mycorrhizal helper bacteria (MHB) in blueberry roots can improve the nutrient structure and physical and chemical properties of blueberry rhizosphere soil by improving the fixation of nitrogen, phosphorus, and potassium in soil and facilitates plant growth and developmental processes by root secretory products such as indole acetic acid (IAA), gibberellin, and growth hormone [34].

Based on the above-mentioned, this study investigated the effects of PFA on the soil environment of blueberry and its soil microorganisms under salt stress through potting experiments to study the effects of the physicochemical properties, soil enzyme activities, and microbial community structure of blueberry inter-root soil, investigate the mechanism of PFA on the growth environment of blueberry under salt stress, and reveal the micro-ecological mechanism of PFA in repairing and improving the salinized soil environment to provide a reference basis for soil improvement to expand the cultivation of blueberry under salt stress.

## 2. Results

### 2.1. Effects of Potassium Fulvic Acid (PFA) on Physical and Chemical Properties and Enzyme Activities of Blueberry Rhizosphere Soil

The effect of PFA on the physicochemical properties of inter-root soil (Table 1). The pH of the soil decreased by 0.12 units in the SS treatment compared with the CK treatment, increased by 0.12 units in the PFA treatment compared with the CK treatment, and increased by 0.21 units in the PFA + SS treatment compared with the SS treatment. Soil EC was elevated by 161.74% in SS treatment compared with CK treatment, there was no significant change in soil EC in PFA treatment compared with CK treatment, and 43.65% reduction in soil EC in PFA + SS treatment compared with SS treatment. In addition, PFA application significantly increased soil OM content, with PFA treatment increasing soil OM content by 16.08% compared with CK treatment, and PFA + SS treatment increasing soil OM value by 9.83% compared with SS treatment. The application of PFA led to a substantial increase in soil AN and AK contents under salt stress conditions. However, this application had no significant impact on soil AP content. (*p* < 0.05).

The following section will examine the effect of PFA on inter-root soil enzyme activities of blueberry (Table 2). As demonstrated in the table, the application of PFA exhibited no statistically significant impact on S-UE activity within the soil matrix. PFA + SS treatment significantly increased S-CAT activity by 15.14% compared with SS treatment. Moreover, SS treatment decreased S-SC activity from 183.05 U/g to 106.54 U/g compared with CK treatment. Whereas, PFA + SS increased S-SC activity by 88.29% from 106.54 U/g to 200.6 U/g compared with SS treatment. A substantial alteration in S-ACP activity was not observed between the PFA and CK treatments. However, the S-ACP activity of the PFA + SS treatment exhibited a significant increase of 13.13% in comparison with the SS treatment (*p* < 0.05).

### 2.2. Comparison of Potassium Fulvic Acid on the Composition and Richness of Bacteria and Fungi in the Rhizosphere Soil of Blueberry

The bacterial 16S rRNA and fungal ITS were subjected to high-throughput sequencing, with the analysis of 16 soil samples from four distinct treatments of the ‘Reka’ blueberry. After further removing the chimeras and short sequences, ASV clustering of the sequences according to the level of 97% similarity yielded 88,885 and 49,134 clean sequences for the bacterial ASVs, and 7987 and 101,950 clean sequences for the fungal ASVs, respectively. The coverage of each bacterial sample was above 97.99%, and the coverage of each fungal sample was above 99.99%. This indicates that the sequencing results are well represented and can truly reflect the sample information.

**Figure 1:**
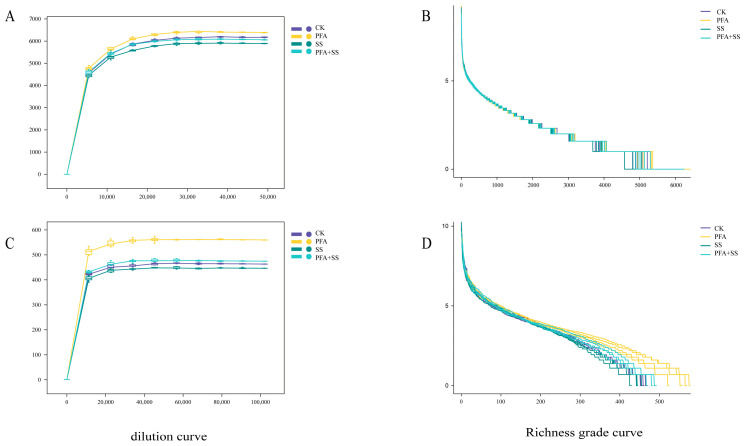
Sparse curve and richness grade curve of bacteria and fungi in the rhizosphere soil of blueberry under different treatments. Note: (**A**,**B**) Bacteria; (**C**,**D**) fungi.

Figure 1 illustrates that the dilution curves for each treated sample reached a stable state, thereby confirming the reliability of the sequencing data. Concurrently, this result indicates that the sequencing depth attained a sufficient level to encompass a broad spectrum of bacterial and fungal groups. This provides a reliable foundation for subsequent analyses of bacterial and fungal community structures, thereby facilitating the continuation of such analyses.

### 2.3. Effect of Potassium Fulvic Acid on the Distribution of Bacterial ASVs in the Inter-Root Soil of Blueberry

**Figure 2:**
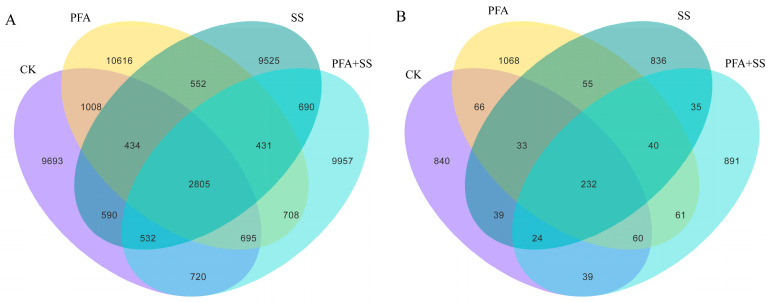
Venn diagram of the ASV distribution of microorganisms in the rhizosphere soil of blueberry. Note: (**A**) Bacteria; (**B**) fungi.

The total number of ASVs in the four treatments was 2805 including 16,477 in the CK treatment, 17,249 in the PFA treatment, 15,559 in the SS treatment, and 16,538 in the PFA + SS treatment (Figure 2A). There were 232 ASVs in the four treatments including 1333 in the CK treatment, 1615 in the PFA treatment, 1294 in the SS treatment, and 1382 in the PFA + SS treatment (Figure 2B). This indicates that the application of PFA could significantly increase the total number of bacteria and fungi in the rhizosphere soil of blueberries.

### 2.4. Analysis of Microorganisms in Rhizosphere Soil of Blueberry (Alpha Diversity)

**Figure 3:**
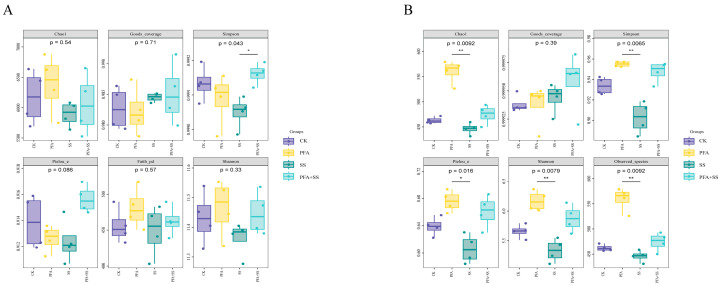
Changes in the α diversity index of microorganisms in the rhizosphere soil of blueberry. Note: (**A**) Bacteria; (**B**) fungi; * *p* < 0.05, ** *p* < 0.01.

The microbial community dynamics of potassium fulvic acid blueberries are shown in Figure 3. The Simpson index was significant in the comparison between the SS treatment and PFA + SS treatment (*p* < 0.05) (Figure 3A). The Chao1 index reflects the total number of colonies in a treatment. Although there was no significant difference in the four treatments, it can be seen that the Chao1 of soil bacteria after adding PFA was higher than that of the CK and SS groups. According to the statistical analysis of fungal α-diversity (Figure 3B), the fungal Chao1 index showed statistically significant differences (*p* < 0.01) between the PFA and SS treatments. (*p* < 0.01). The Simpson index, Pielou’s index, Shannon index, and Observed_species index between the SS treatment and PFA treatment were extremely significant (*p* < 0.01), indicating that the addition of PFA and NACl could significantly change the diversity of fungal communities in the soil, and the application of PFA could significantly increase the diversity of fungi in the community.

### 2.5. Effects of Potassium Fulvic Acid on PCoA of Bacterial and Fungal Communities in Rhizosphere Soil of Blueberry and Analysis of Differences Between Groups

**Figure 4:**
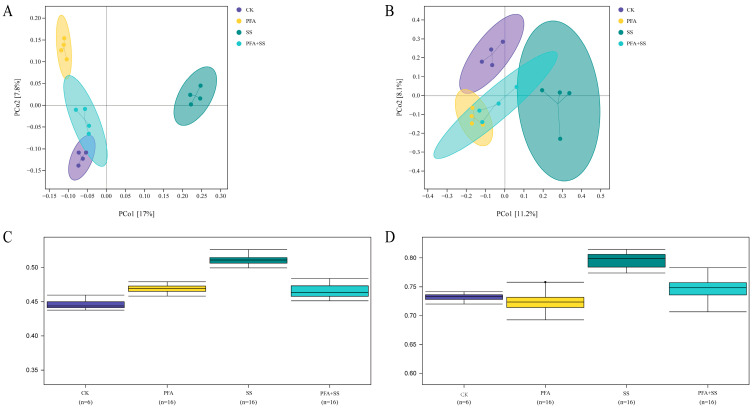
Analysis of PCoA and inter-group differences of microbial community in the rhizosphere soil of blueberry under different treatments. Note: (**A**,**C**) Bacteria; (**B**,**D**) fungi.

Based on the Bray–Curtis distance, the data in the sample were simplified for PCoA and inter-group difference analysis (Figure 4). PCoA analysis showed that the composition of the rhizosphere bacterial and fungal communities in the four treatments was distributed in different quadrants (Figure 4A,B). PCo1 explained 17% and 11.2% of the variance variables, and PC2 explained 7.8% and 8.1% of the variance variables, respectively. Differences between groups were analyzed by the PERMANOVA test (Figure 4C,D). From the difference analysis diagram between groups, it can be seen that there were differences in the microorganisms between the SS group and the other groups.

### 2.6. Effect of Potassium Fulvic Acis on Bacterial Community Composition in Blueberry Rhizosphere Soil

#### 2.6.1. Analysis of Microbial Community at the Phylum Level

**Figure 5:**
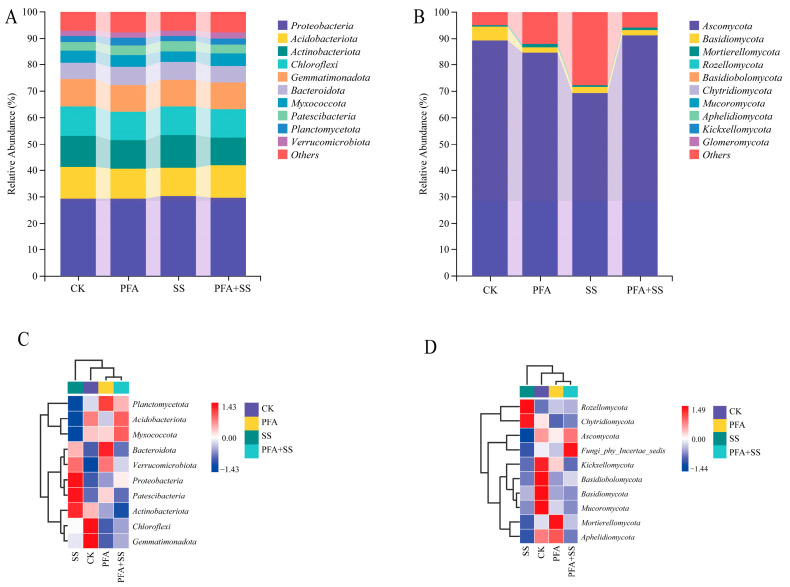
The relative abundance histogram and species distribution heat map of microbial community at the phylum level. Note: (**A**,**C**) Bacteria; (**B**,**D**) fungi.

The results of high-throughput sequencing revealed the presence of 38 phyla, 112 classes, 253 orders, 359 families, and 491 genera of bacteria in the rhizosphere soil of blueberries under various treatments. Similarly, a total of 8 phyla, 17 classes, 40 orders, 70 families, and 120 genera were identified in the rhizosphere soil fungi. The distribution of bacteria at the phylum level in the different treatment groups is shown in Figure 5A including Proteobacteria, Acidobacteria, Actinobacteria, Chloroflexi, Gemmatimonadota, Bacteroidetes, Myxococcota, and Patescibacteria. Planctomycetota and Verrucomicrobiota were the dominant phyla, with a richness of more than 1%. The average relative abundances were 29.68%, 11.63%, 11.26%, 10.87%, 10.08%, 6.65%, 4.46%, 3.48%, 2.41%, and 2.00%, respectively, and these ten phyla accounted for 92.50% of the total richness. The distribution of fungi in different treatments at the phylum level (Figure 5B) showed that Ascomycota, Basidiomycota, and Mortierellomycota were the dominant phyla with a richness of >1%, and the average relative abundances were 83.64%, 2.88%, and 0.82%, respectively.

From the heat map of the phylum-level species distribution of the soil bacteria and fungi (Figure 5C,D), the abundance of Chloroflexi, Gemmatimonadota, and Acidobacteriota in the CK treatment was high. The abundances of Proteobacteria, Patescibacteria, and Actinobacteria were high in the SS treatment. The abundance of Planctomycetota and Bacteroidetes was high in PFA. The abundance of Acidobacteria and Myxococcota was high in the PFA + SS treatment.

As shown in Figure 5D, the abundance of Kickxellomycota, Basidiobolomycota, Basidiomycota, and Mucoromycota was high in the CK treatment. The abundances of Rozellomycota, and Chytridiomycota were high in the SS treatment, while the abundances of Mortierellomycota and Aphelidiomycota were high in the PFA treatment. The abundance of Ascomycota and Fungi_phy_Incertae_sedis was high in the PFA + SS treatment.

#### 2.6.2. Composition of Bacterial Community at Genus Level

**Figure 6:**
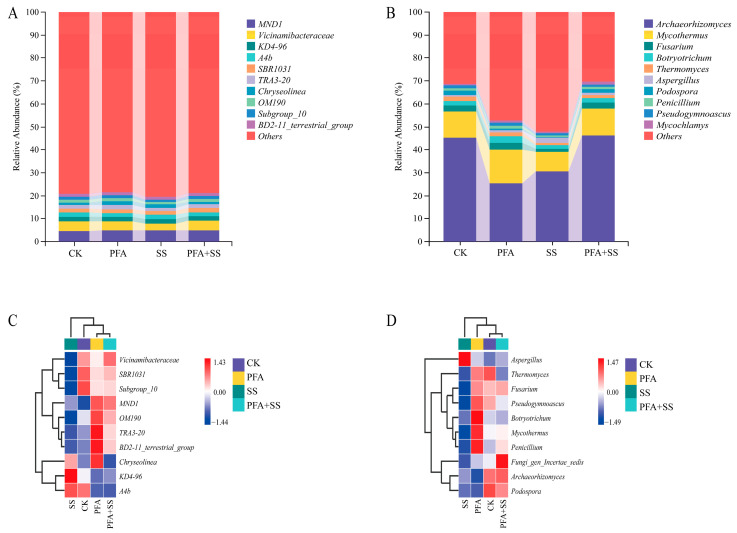
The relative abundance histogram and species distribution heat map of the microbial community at the genus level. Note: (**A**,**C**) Bacteria; (**B**,**D**) fungi.

The top 10 bacterial colonies at the genus level were selected for comparative analysis at different blueberry fruit developmental stages (Figure 6A). Among them, *MND1*, *Vicinamibacteraceae*, *KD4-96*, *A4b*, *SBR1031*, *TRA3-20*, *Chryseolinea*, *OM190*, and *Subgroup_10*, *BD2-11_terrestrial_group* were the dominant bacterial genera, accounting for 4.88%, 3.83%, 1.85%, 1.81%, 1.73%, 1.66%, 1.29%, 1.26%, 1.19%, and 1.18% of the total abundance, respectively.

The top 10 fungal colonies at the genus level were selected for comparative analysis (Figure 6B). *Archaeorhizomyces*, *Mycothermus*, *Fusarium*, *Botryotrichum*, *Thermomyces*, *Aspergillus*, *Podospora*, *Penicillium*, *Pseudogymnoascus*, and *Mycochlamys* were the dominant fungal genera, accounting for 36.97%, 11.46%, 2.39%, 2.12%, 1.39%, 1.32%, 1.14%, 1.05%, 1.04%, and 0.99% of the total abundance, respectively.

According to the analysis of the genus-level species distribution heat map of soil bacteria and fungi (Figure 6C,D), the abundances of bacteria *A4b*, *Vicinamibacteraceae*, *SBR1031*, and *Subgroup_10* were high in the CK treatment. The abundance of bacteria *A4b*, and *KD4-96* was high in the SS treatment group. The bacterial genera *Chryseolinea*, *MND1*, *OM190*, *TRA3-20*, and *BD2-11_terrestrial_groups* were abundant in the PFA treatment. The abundance of *Vicinamibacteraceae*, and *MND1* was high in the PFA + SS treatment.

As shown in Figure 6D, the abundances of *Archaeorhizomyces*, *Podospora*, and *Thermomyces* were high in the CK treatment. The abundance of the fungal genera *Aspergillus* was high in the SS treatment. The abundances of *Botryotrichum*, *Mycothermus*, and *Penicillium* were high in the PFA treatment, and the abundances of *Archaeorhizomyces*, *Podospora*, and *Fungi_gen_Incertae_sedis* were high in the PFA + SS treatment.

### 2.7. Correlation Analysis Between Bacterial and Fungal Communities in Rhizosphere Soil of Potassium Fulvic Acids Blueberry and Soil Environmental Factors

**Figure 7:**
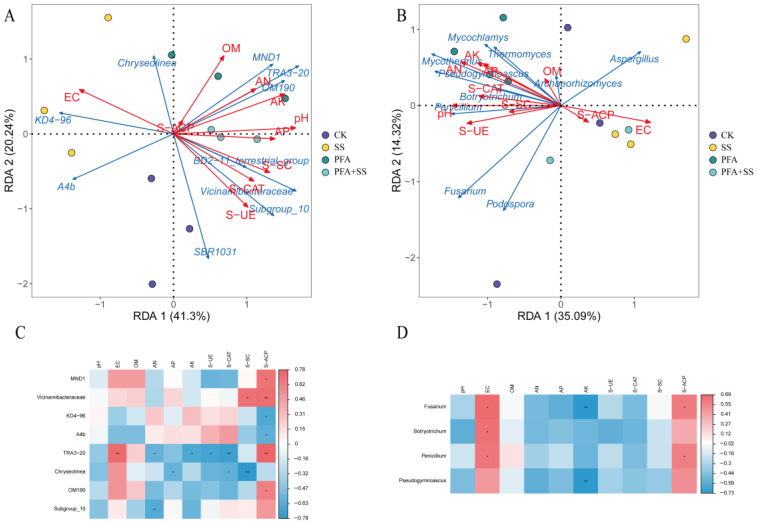
RDA analysis and correlation analysis between the top ten dominant bacteria and fungi and soil environmental factors. Note: (**A**,**C**) Bacteria; (**B**,**D**) fungi. * *p* < 0.05, ** *p* < 0.01.

Redundancy analysis was performed between 10 dominant bacteria and fungi in soil and 10 environmental factors in soil (Figure 7). It can be seen from Figure 7A,B that the relationship between each treatment and the soil environmental factors and soil microbial community. The first and second axes of the bacterial RDA analysis were 41.3% and 20.24% (Figure 7A), respectively, and the total interpretation rate was 61.54%. Except for the EC value, *KD4-96*, *A4b*, and *Chryseolinea* were positively correlated with the SS treatment, whereas other environmental factors and dominant bacteria were positively correlated with the PFA and PFA + SS treatments. It can be seen that *KD4-96* and *A4b* belong to the salt-tolerant bacteria. The first and second axes of the RDA analysis of soil fungi were 35.09% and 14.32% (Figure 7B), respectively, and the total interpretation rate reached 49.41%. The diagram shows that *Aspergillus* was positively correlated with SS treatment, indicating that *Aspergillus* is a salt-tolerant fungal genus. The other dominant fungal genera were also positively correlated with the PFA and PFA + SS treatments. As illustrated by the correlation heat map, a statistically significant positive correlation was observed between *TRA3-20* in bacteria (Figure 7C) and EC and S-ACP (*p* < 0.01). There was a significant negative correlation between the *TRA3-20* and S-CAT scores (*p* < 0.01). *Chryseolinea* was significantly negatively correlated with the S-SC (*p* < 0.01). *Vicinamibacteraceae* was significantly positively correlated with S-ACP (*p* < 0.01). *Fusarium* and *Pseudogymnoascus* fungi (Figure 7D) were significantly negatively correlated with AK (*p* < 0.01). The soil pH and OM had no significant effect on the dominant bacteria, whereas *Fusarium*, *Botryotrichum*, *Penicillium*, and *Pseudogymnoascus* were significantly affected by EC, AK, and S-ACP in the soil, and the other soil physical and chemical indicators were not significant.

### 2.8. Effects of Potassium Fulvic Acid on the Growth and Physiology of Blueberry

It can be seen from Table 3 that there was no significant difference in the stem diameter between the other treatments, except that the stem diameter of blueberry increased significantly under the PFA + SS treatment. Furthermore, in comparison with the CK treatment, the SS treatment resulted in a 27.04% decrease in plant height, a 25.82% decrease in total chlorophyll content, and a 36.70% decrease in root activity. In comparison with the SS treatment, the PFA + SS treatment resulted in an increase in plant height of 26.53%, a rise in the total chlorophyll content of 23.70%, and an enhancement in the root activity of 23.26%. However, there was no significant difference in the growth and physiological indices of blueberry between the CK and PFA treatments.

## 3. Discussion

### 3.1. Effects of Potassium Fulvic Acid on Physical and Chemical Properties and Soil Enzyme Content of Blueberry Rhizosphere Soil

In recent years, soil nutrients have been found to be beneficial to the normal growth and stress resistance of plants. An environment suitable for plant growth can promote the absorption of nutrients in the soil by plants, and the soil EC value can reflect the concentration of soluble salt. A high EC value represents a high salt content in the soil, which is not conducive to plant growth [35,36]. In this study, bilberry was suitable for growth in an environment with a pH value of 4.5–5.5 [37]. The addition of PFA did not significantly change the soil pH value, indicating that the appropriate addition of PFA would not put blueberry in an unsuitable growth environment. The addition of PFA in the normal environment did not increase the EC value, while the addition of PFA in the salt environment significantly decreased the EC value in the soil, indicating that PFA can reduce the salinity in the soil, which is consistent with the results of Sun et al. [35] and Zhang et al. [27]. In comparison with CK and SS, the incorporation of potassium fulvic acid has been demonstrated to result in a substantial augmentation in the content of organic matter, alkali-hydrolyzable nitrogen, and available potassium in soil. Potassium fulvic acid has been found to contain elevated levels of carbon and potassium, which can be utilized as a carbon source to directly supplement soil organic matter and thereby increase its content. The process has the capacity to supply soil with potassium, which is readily absorbed by plants. Furthermore, it has the capability to counteract the deleterious effects of elevated sodium concentrations in soil on plant life, which is consistent with the results of Li et al. [38] and Gao [39]. However, the results demonstrated that the impact on the available phosphorus (AP) content in the soil was not statistically significant. This outcome may be attributed to the potential of FA in reducing soil phosphorus fixation, modulating soil phosphorus migration, and enhancing the transportation of phosphorus to blueberry roots [40]. At the same time, the mycorrhiza of blueberries could also promote the absorption of P by blueberries, so the content of available phosphorus in soil does not change significantly [33,34].

Soil enzymes are important for improving the soil nutrient structure. Urease (S-UE) is an enzyme that plays a pivotal role in the conversion of urea. It is intimately associated with the nitrogen cycle within soil ecosystems [41]. The widespread presence of sucrase (S-SC) in the soil is closely related to the soil carbon cycle [42]. Phosphatase (S-ACP) catalyzes the mineralization of soil organic phosphorus compounds and is closely related to the soil phosphorus cycle [43]. Catalase (S-CAT) has been identified as a significant indicator of the soil microecological environment. Its relationship to the intensity of soil respiration and the activity of soil microbes has been well-documented [44]. The test results showed that with SS treatment, the PFA + SS treatment significantly increased the activity of S-UE, promoted the transformation of N in soil, increased the content of N in soil, and significantly reduced the loss of N in salt-stressed soil, which is consistent with the study by Sun [45]. The addition of PFA alone even reduced the S-SC activity in the soil. This may be because PFA has its own carbon source that is applied to the soil. The soil organic carbon increased significantly, and more organic carbon was added to the soil carbon cycle, which inhibited S-SC activity. However, the application of PFA in a saline environment can significantly increase the activity of S-UE, S-ACP, S-SC, and S-CAT, which is consistent with the results of Zhang et al. [27]. The experiment demonstrated that PFA significantly enhanced the ability of plants to thrive in salt-stressed environments and alleviated the nutrient deficiency of plants in such conditions by increasing the activity of various enzymes.

### 3.2. Effects of Potassium Fulvic Acid Treatment on Rhizosphere Soil Microbial Diversity and Composition in Blueberry

The results demonstrated that salt stress significantly reduced the diversity of the soil microbial community. However, the application of PFA was found to promote the diversity of the microbial community in terms of both the quantity and structure. This finding is consistent with the observations reported by Liu [46]. In previous studies, Proteobacteria, Acidobacteria, and Actinobacteria were identified as the predominant bacterial phyla in the soil microorganisms of blueberries. The number of communities in the critical period of blueberry growth increased significantly, which can enhance the plant uptake of soil nutrients effectively. [32,47]. Previous studies have found that Proteobacteria has the function of enriching carbon in the soil and can adapt to various complex environments, which plays an important role in improving soil organic carbon [48,49]. Acidobacteria can decompose plant and animal residues, thereby increasing the soil organic matter content [50]. In addition, these dominant phyla are also abundant in the soil of other plants, which play a role in maintaining and promoting plant growth and protecting plants from external abiotic stress [40] such as citrus [27], grape [51], and wheat [45]. In this experiment, in addition to Proteobacteria, Acidobacteria, Actinobacteria, Chloroflexi, Gemmatimonadota, Ascomycota, and Basidiomycota were found, and Proteobacteria, Patescibacteria, and Actinobacteria were highly abundant, which was similar to the results of Zhang et al. [49]. Shi et al. [52] found that the abundance of Acidobacteriota and Myxococcota decreased in salt-stressed soil, and the same results were obtained in this experiment. However, the addition of PFA in salt-stressed environments could increase the abundance of Acidobacteriota and Myxococcota, indicating that the addition of PFA could adjust the soil bacterial community structure. Regarding the change in fungal phylum, the addition of PFA in the salt environment can increase the abundance of Ascomycota and Fungi_phy_Incertae_sedis, Among them, Ascomycota has an effect on the carbon cycle in soil and can be used as an index to measure the accumulation of soil organic carbon to a certain extent [53]. In general, the application of PFA in blueberry soils can significantly affect the number and composition of the soil microbial communities.

### 3.3. Effects of Potassium Fulvic Acid on the Growth and Physiological Activity of Blueberry

Research has demonstrated that the incorporation of potassium fulvic acid into a standard environment exerts a minimal influence on the growth and physiological indices of blueberry plants. Nevertheless, the utilization of potassium fulvic acid within a saline environment has been demonstrated to enhance the growth of blueberries. This improvement in growth activity has been observed to be concomitant with an enhancement in root activity and the promotion of chlorophyll synthesis under salt stress conditions. These observations are analogous to those reported in the study conducted by Zhang et al. [54]. In this study, it was found that there was still a certain gap between the growth status of blueberry seedlings under the salt stress environment and the growth status of blueberry seedlings under the normal environment. Combined with the analysis of soil environmental indicators, we found that potassium fulvic acid could improve the microbial structure in the soil as well as the physical and chemical indicators of the soil environment. However, the excess Na^+^ in the soil was not completely eliminated during the test period, which also indicates that soil improvement is a long-term process. It takes a long time to apply modifiers to achieve the best improvement effect.

The study indicated that the vast majority of bacterial and fungal microorganisms were negatively correlated with salt stress. In addition to the EC value and S-ACP, other soil physical and chemical indicators and enzyme activity were positively correlated with the application of PFA treatment. Through RDA analysis, it was found that whether it was the soil nutrients, soil enzymes, or dominant bacteria, it was closer to the treatment with PFA, indicating that the change in soil nutrients directly affects the community structure and function of rhizosphere microorganisms. Changes in the microbial community structure can be fed back to plant growth, thereby promoting the growth of blueberries. [27]. In this study, there was a strong correlation between the PFA effects on the blueberry soil microenvironment and the changes in soil nutrients and microbial community structure.

## 4. Materials and Methods

### 4.1. Overview of Test Site

This experiment was carried out in the greenhouse (E120°24′, N43°48′) of the blueberry industrialization innovation scientific research practice base of Jilin Agricultural University. The test time was from June to August 2024. Potassium fulvic acid (PFA) was procured from Shanghai McLean Biochemical Technology Co. Ltd. (Shanghai, China), and NaCl was purchased from Tianjin Tianli Chemical Reagent Co. Ltd. (Tianjin, China). The soil matrix was garden soil:peat soil:perlite mixed in a volume ratio of 2:1:1, and the pH was adjusted by sulfur powder to maintain the soil pH at about 5.0. The ambient temperature was maintained within the range of 25 ± 3 °C, and the humidity level was maintained within the range of 50–70%.

### 4.2. Experimental Design and Sample Collection

Two-year-old ‘Reka’ blueberry seedlings grown under the same conditions were transferred to plastic pots filled with soil. Each blueberry test seedling was planted in a flower pot (with an external diameter measuring 21 cm, internal diameter of 17.7 cm, height of 12 cm, and diameter at the base of 11.5 cm, accompanied by a tray). After transplanting, the seedlings were slowed down for 30 days, and water was used for irrigation during the period. After the seedlings were slowed down, four treatments were performed on the seedlings: only water treatment (CK); fulvic acid potassium solution (60 mg·L^−1^) (PFA); NaCl solution (120 mmol·L^−1^ Na^+^) (SS); potassium fulvic acid solution (60 mg·L^−1^); and NaCl solution (120 mmol·L^−1^ Na^+^) were mixed as the PFA + SS treatment 3 times, and each pot of blueberry was irrigated with 500 mL of the treatment solution (NaCl and PFA mixture), divided into 5 times, and irrigated for 5 days. Three days after treatment, water was poured once to ensure the normal water demand of plants. Each treatment comprised 10 seedlings, yielding a total of 40 seedlings that were utilized as three biological replicates. Rhizosphere soil was collected 30 days after treatment.

Plant samples and mature leaves were collected and put in a refrigerator at 4 °C for backup. The root activity was measured after the rhizosphere soil was collected. The collection of soil samples was conducted by first extracting the soil surrounding the root system into a sterile self-sealing bag. This was achieved by vigorously shaking the root system after removing a substantial portion of the soil. The residual soil that remained unshaken was then brushed away using a sterile brush. It is imperative to note that the brush must undergo a thorough cleansing, disinfection, and drying process after each use to ensure optimal hygiene and effectiveness. The final stage of the procedure involved the removal of the residual root system from the soil sample, after which the impurities were subjected to a sieve with a pore size of 2 mm. The soil sample was stored in a refrigerator set at 4 °C for the purpose of determining its physical and chemical properties as well as its enzyme activity. The high-throughput sequencing soil sample was placed in a refrigerator set at −80 °C for subsequent use.

### 4.3. Determination of Plant Growth and Physiological Indices

The plant height and stem diameter were measured using a soft ruler and a vernier caliper, respectively. The chlorophyll content was measured using the ethanol-acetone mixed solution immersion method, and the root activity was measured using the triphenyl tetrazolium chloride (TTC) method [55].

### 4.4. Determination of the Physicochemical Indices and Enzyme Activities of Rhizosphere Soil

The determination methods of physical and chemical indexes of rhizosphere soil are shown in Table 4.

In accordance with the manufacturer’s guidelines, enzyme activity detection kits were utilized for the extraction and quantification of catalase (S-CAT), acid phosphatase (S-ACP), sucrase (S-SC), and urease (S-UE) in the soil. This was achieved by employing distinct enzyme activity detection kits (Beijing Soleibao Technology Co. Ltd., Beijing, China), and the activities of these enzymes were subsequently measured using a microplate reader.

### 4.5. Microbial Community Analysis of Rhizosphere Soil Samples

The detailed methodology for the blueberry rhizosphere microbiome analysis is as follows:

(1) Using an OMEGA Soil DNA Kit (D5635-02) (Omega Bio-Tek, Norcross, GA, USA), the total microbial DNA was extracted.
(2) PCR amplification was performed using qualified soil DNA as a template. The target fragments were the V3–V4 region of the bacterial 16S rRNA gene and fungal ITS1. The ITS1 was selected. The upstream and downstream primers were 338F, 806R, ITS5, and ITS2. The same amplification system (25 μL) for bacteria and fungi: Q5 high-fidelity DNA polymerase 0.25 μL, 5 × reaction buffer 5 μL, 5 × GC buffer 5 μL, dNTP (10 mM) 2 μL, forward primer (10 μM) 1 μL, reverse primer (10 μM) 1 μL, DNA template 2 μL.
(3) The amplification conditions were as follows: 98 °C for 5 min, 98 °C for 30 s, 55 °C for 30 s, and 72 °C for 45 s, a total of 30 cycles; the reaction was extended at 72 °C for 5 min, and the reaction was terminated at 12 °C.
(4) The PCR amplification products were detected using 0.8% agarose gel electrophoresis, and the qualified products were entrusted to Shanghai Pasenuo Biotechnology Co. Ltd. (Shanghai, China) for sequencing and microbial diversity analysis using the Illumina NovaSeq platform (Shanghai, China).

### 4.6. Statistical Analysis

The experimental data were analyzed using Excel 2019 and IBM SPSS Statistics 26 for one-way analysis of variance (ANOVA) to analyze the differences between the values of each group.

Alpha diversity analysis was performed using QIIME2 2022.11 software to calculate the beta diversity distance matrix. Based on the weighted UniFrac distance, redundancy analysis (RDA) was performed using R (v3.6.0) software, and the species composition histogram was analyzed.

## 5. Conclusions

The findings of this study demonstrated that potassium fulvic acid exhibited the capacity to enhance the growth and physiological activity of blueberry plants, promote the growth of blueberry under salt stress, increase root activity, and promote chlorophyll synthesis in the leaves. Concurrently, substantial variations in the soil physical and chemical properties were observed among the various treatments. The application of PFA has been demonstrated to have the capacity to enhance the content of soil OM, reduce the EC value in salt environments, and augment the content of soil AN and AK. It also affects the soil enzyme activity. Salt treatment inhibits enzyme activity in soil, and the application of PFA to saline environments can effectively improve the soil enzyme activity. In addition, PFA also improved the bacterial and fungal community structure in blueberry soil and increased the richness of Acidobacteriota, Myxococcota, Ascomycota, and Fungi_phy_Incertae_sedis compared with the salt stress environment.

Potassium fulvic acid has been demonstrated to have a significant impact on the growth environment of blueberry plants. The primary mechanism through which it exerts its effect involves a reduction in the electrical conductivity (EC) value of the soil, an increase in the content of soil organic matter, AN, and AK, and a consequent alteration in the structure of the soil microbial community. Increasing these microbial communities can increase the enzyme activity in the soil and improve nutrient uptake by the blueberry roots. This can reduce the damaging effects of salt stress on blueberry plants. Therefore, PFA has a significant effect as a soil amendment for improving the saline cultivation environment of blueberry.

## Figures and Tables

**Table 1:** Analysis of the physicochemical properties of blueberry rhizosphere soil.

Treatment	pH	EC (uS/cm)	OM (g/kg)	AN (mg/kg)	AP (mg/kg)	AK (mg/kg)
CK	4.84 ± 0.04 b	697 ± 59 c	16.80 ± 0.29 c	150 ± 5 b	33.87 ± 1.12 a	305 ± 7 c
SS	4.72 ± 0.04 c	1824 ± 159 a	17.80 ± 0.31 b	145 ± 5 b	29.37 ± 1.70 b	268 ± 4 d
PFA	4.96 ± 0.04 a	698 ± 15 c	18.34 ± 0.51 b	169 ± 4 a	35.40 ± 1.43 a	425 ± 10 a
PFA + SS	4.93 ± 0.05 a	1028 ± 7 b	19.55 ± 0.19 a	160 ± 5 a	35.67 ± 1.23 a	399 ± 17 b

Notes: CK, seedlings without PFA (fulvic acids potassium) and salt stress (control group); PFA, seedlings treated with PFA without salt stress; SS, seedlings under salt stress; PFA + SS, seedlings treated with PFA and salt stress simultaneously. As indicated by the letters “a”, “b”, “c” and “d”, there are statistically significant differences between treatments at the 0.05 *p*-value cutoff.

**Table 2:** Analysis of enzyme activities in the rhizosphere soil of blueberry.

Treatment	S-UE Activity (U/g)	S-CAT Activity (U/g)	S-SC Activity (U/g)	S-ACP Activity (U/g)
CK	1585 ± 64 a	34 ± 1 a	183 ± 17 ab	798 ± 18 c
SS	1104 ± 76 b	26 ± 1 c	107 ± 25 c	904 ± 21 b
PFA	1470 ± 141 a	33 ± 0 a	161 ± 12 b	773 ± 6 c
PFA + SS	1406 ± 79 a	30 ± 0 b	201 ± 12 a	1023 ± 84 a

Note: S-UE, soil urease; S-CAT, soil catalase; S-SC, soil sucrase; S-ACP, soil acid phosphatase. As indicated by the letters “a”, “b”and “c”, there are statistically significant differences between treatments at the 0.05 *p*-value cutoff.

**Table 3:** Effects of different treatments on the growth and physiology of blueberry.

Treatment	Plant Height (mm)	Stem Thick (mm)	Total Chlorophyll Content (mg/g)	Root Activity [ug/(g·h)]
CK	351 ± 12 a	2.63 ± 0.17 b	1.82 ± 0.05 a	235 ± 5 a
SS	256 ± 5 c	2.47 ± 0.09 b	1.35 ± 0.04 c	149 ± 7 c
PFA	351 ± 14 a	2.65 ± 0.46 b	1.78 ± 0.03 a	226 ± 5 a
PFA + SS	324 ± 3 b	3.21 ± 0.03 a	1.67 ± 0.05 b	184 ± 2 b

As indicated by the letters “a”, “b” and “c”, there are statistically significant differences between treatments at the 0.05 *p*-value cutoff.

**Table 4:** Measured parameters and determination method.

Measured Parameters	Determination Method [56]
Electrical conductivity (EC) and pH	Measured at a water/soil mass ratio of 5:1.
Organic matter (OM) content	Used the high-temperature external thermal potassium dichromate oxidation-volumetric method.
Alkali-hydrolyzable nitrogen (AN) content	Used the alkali solution diffusion absorption method.
Content of available phosphorus (AP)	The 0.5 mol/L NaHCO_3_ extraction-molybdenum antimony colorimetric method.
Content of available potassium (AK)	Used a 1 mol/L NH_4_OAc extraction-flame photometric method.

## Data Availability

The datasets generated during and/or analyzed during the current study are available from the corresponding author on reasonable request.
